# New sesquiterpene hydroquinones from the Caribbean sponge *Aka coralliphagum*

**DOI:** 10.3762/bjoc.10.52

**Published:** 2014-03-06

**Authors:** Qun Göthel, Matthias Köck

**Affiliations:** 1 Alfred-Wegener-Institut, Helmholtz-Zentrum für Polar- und Meeresforschung, Am Handelshafen 12, 27570 Bremerhaven, Germany

**Keywords:** *Aka coralliphagum*, bioassay, natural products, NMR, sesquiterpene hydroquinone, structure elucidation

## Abstract

Four new sulfated sesquiterpene hydroquinones siphonodictyals E1–E4 (**1**–**4**) and cyclosiphonodictyol A (**5**) were isolated from a sample of the Caribbean sponge *Aka coralliphagum* collected off the coast of San Salvador in the Bahamas. The structures of the new compounds were elucidated on the basis of mass spectrometric and NMR spectroscopic analysis. Compounds **1**–**4** are derivatives of siphonodictyal E (**9**). Siphonodictyal E4 (**4**) exhibited mild antiproliferation activity against L929 mouse fibroblast, KB-31 epidermoid carcinoma, and MCF-7 breast cancer cell lines, while siphondictyal E3 (**3**) and cyclosiphonodictyol A (**5**) showed moderate activity against Gram-positive bacteria.

## Introduction

*Aka coralliphagum* (*Siphonodictyon coralliphagum*) is known to have four distinct morphological forms: forma *typica*, f. *tubulosa*, f. *obruta*, and f. *incrustans* [1]. This sponge has the ability to burrow into live coral heads, leaving only the oscular chimney protruding (*typica*) or the flat crusts (*incrustans*) exposed. The oscular chimneys or the flat crusts are encircled by a so-called "dead zone" which protects the sponge from overgrowth [2]. Sullivan and Faulkner have proposed that the coral polyps are killed by the viscous mucus containing the toxic secondary metabolites which are produced by the sponge tissue [3]. This ecological observation inspired the chemical investigation of this sponge by different research groups. Thus many secondary metabolites have been reported, such as the sesquiterpene phenolic aldehydes, siphonodictyals B, C, D, E, the monosulfated siphonodictyols G and H, the disulfated siphonodictyal B3, and siphonodictyoic acid [2,4–5]. The sponge under our investigation is of the growth form *incrustans*, which was collected off the coast of San Salvador in the Bahamas. Fractionation of the aqueous *n*-BuOH extract yielded the new compounds siphonodictyals E1–E4 (**1**–**4**) and cyclosiphonodictyol A (**5**). In this article, we describe the isolation, structural elucidation, and bioactivity of compounds **1**–**5** from the *Aka* growth form *incrustans*, as well as discuss the biosynthetic pathway of related compounds.

## Results and Discussion

**Figure 1:**
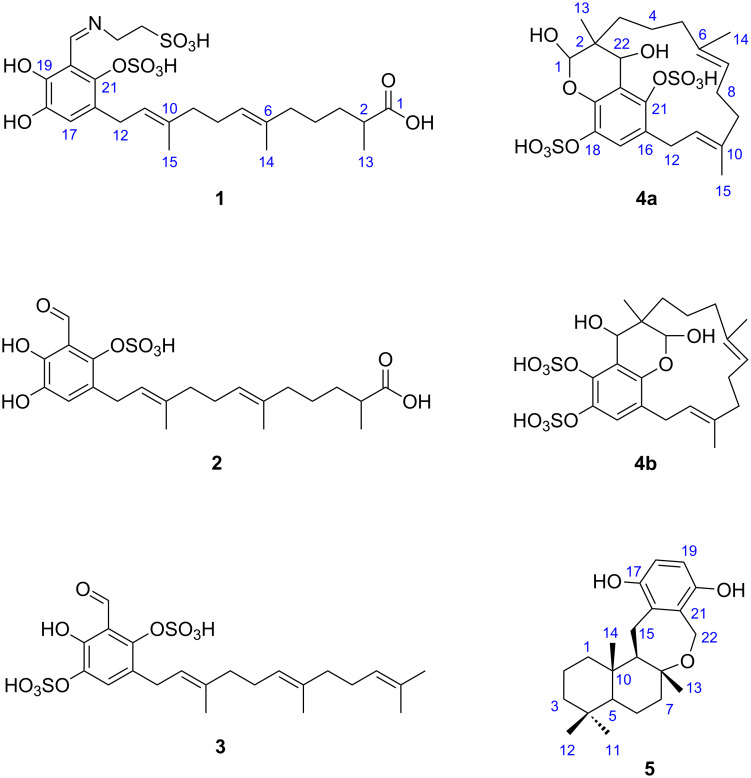
Structures of the new compounds siphonodictyals E1–E4 (**1**–**4**) and cyclosiphonodictyol A (**5**) isolated from the sponge *Aka coralliphagum*. Since the structure of siphonodictyal E4 (**4**) could not be unambiguously assigned, the two possible constitutional isomers (**4a** and **4b**) are shown.

The aqueous *n*-BuOH extract from *Aka coralliphagum* was subjected to solvent partitioning, followed by gel chromatography and reversed phase (C18) HPLC to yield siphonodictyals E1–E4 (**1**–**4**) and cyclosiphonodictyol A (**5**) (Figure 1). The structure elucidation was based on 1D and 2D NMR as well as HRMS-ESI(−) experiments. The ^1^H and ^13^C NMR chemical shifts for the five new compounds are given in Table 1.

**Table 1:** NMR data of compounds **1**–**5** (δ_C_ 150 MHz, δ_H_ 600 MHz in DMSO-*d*_6_).^a^

No.	**1**		**2**		**3**		**4**		**5**	

	δ_C_	δ_H_	δ_C_	δ_H_	δ_C_	δ_H_	δ_C_	δ_H_	δ_C_	δ_H_

1	177.8	–	177.5	–	25.5	1.62 (s)	96.2	5.42 (s)	39.4	0.76 (m)
										1.91 (m)
2	38.7	2.28 (m)	38.6	2.28 (m)	130.6	–	37.3	–	18.2	1.38 (m)
										1.60 (m)
3	32.9	1.25 (m)	32.8	1.25 (m)	124.2	5.05 (t, 7.1)	31.4	1.03 (m)	41.6	1.08 (dt, 4.0, 13.5)
		1.47 (m)		1.47 (m)				1.13 (m)		1.33 (m)
4	24.9	1.32 (m)	24.9	1.31 (m)	26.3	2.00 (m)	22.1	0.56 (m)	33.1	–
								1.11 (m)		
5	38.9	1.91 (m)	39.4	1.91 (t, 6.9)	39.3	1.92 (m)	39.3	1.28 (m)	55.4	0.83 (m)
								1.75 (m)		
6	134.4	–	134.4	–	134.4	–	134.9	–	19.8	1.58 (m)
										1.60 (m)
7	124.0	5.09 (t, 6.5)	124.0	5.09 (t, 6.8)	124.0	5.09 (t, 7.1)	121.7	4.52 (t, 5.9)	40.1	1.43 (m)
										1.60 (m)
8	26.2	2.06 (m)	26.2	2.06 (m)	26.2	2.06 (m)	23.8	1.89 (m)	78.5	–
								2.01 (m)		
9	40.1	1.98 (m)	39.8	1.99 (t, 7.2)	40.1	1.97 (m)	39.0	1.92 (m)	58.1	1.32 (m)
10	134.5	–	135.4	–	135.5	–	131.9	–	38.2	–
11	123.7	5.19 (t, 7.2)	122.9	5.21 (t, 7.2)	122.6	5.21 (t, 7.0)	125.7	5.10 (t, 7.9)	33.1	0.83 (s)
12	27.2	3.25 (d, 3.4)	26.9	3.33^b,c^	27.1	3.35^b,c^	29.8	2.58 (dd, 6.8, 12.6)	21.2	0.78 (s)
								3.99 (dd, 9.6, 12.4)		
13	17.0	1.01 (d, 6.6)	17.0	1.01 (d, 6.9)	17.6	1.54 (s)	19.9	1.04 (s)	21.6	1.31 (s)
14	15.7	1.53 (s)	15.6	1.53 (s)	16.0	1.55 (s)	17.2	1.36 (s)	15.3	0.79 (s)
15	15.9	1.65 (s)	15.9	1.66 (s)	15.8	1.66 (s)	15.1	1.62 (s)	20.8	2.33 (dd, 9.6, 15.5)
										3.06 (d, 15.5)
16	120.5	–	125.9	–	125.5	–	126.5	–	129.9	–
17	117.7	6.50 (s)	122.8	6.84 (s)	129.5	7.48 (s)	120.8	7.19 (s)	146.4	–
18	144.0	–	141.8	–	137.7	–	137.2	–	113.7	6.45 (d, 8.6)
19	156.4^c^	–	147.8	–	151.3	–	143.2	–	112.4	6.38 (d, 8.6)
20	110.9^c^	–	115.5	–	115.9	–	120.3	–	146.5	–
21	141.8^c^	–	144.5	–	148.2	–	142.9	–	127.7	–
22	164.6	8.67 (s)	197.2	10.10 (s)	196.6	10.11 (s)	65.3	4.44 (s)	56.7	4.50 (d, 15.0)
										4.64 (d, 15.0)
23	51.2^d^	3.83 (t, 6.5)								
24	51.2^d^	2.82 (t, 6.2)								
1-OH								7.07 (bs)		
18-OH				9.09						
19-OH				11.37						
22-OH								5.09 (bs)		

^a^δ are given in ppm (reference: DMSO-*d*_6_, 2.50 ppm, 39.52 ppm), coupling constants are given in Hz; assignments were confirmed by 2D NMR experiments (COSY, HSQC, HMBC). ^b^Overlap with water peak. ^c^Chemical shifts were obtained from HMBC spectra. ^d^Overlap with each other.

**Figure 2:**
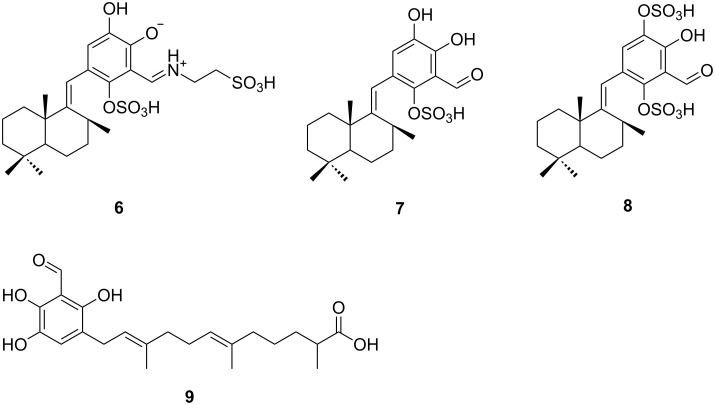
Structures of the related known compounds siphonodictyal B1 (**6**), siphonodictyal B2 (**7**), siphonodictyal B3 (**8**), and siphonodictyal E (**9**) from the sponge *Aka coralliphagum*.

**Figure 3:**
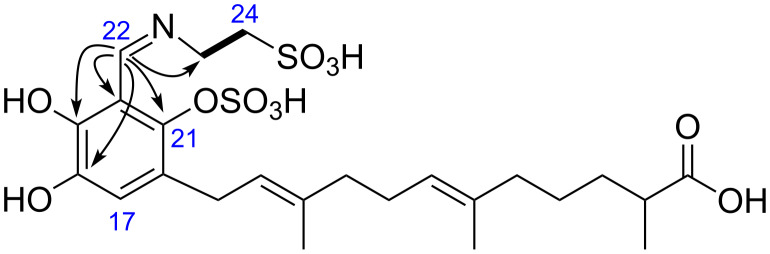
Selected ^1^H,^13^C-HMBC correlations (H → C) and ^1^H,^1^H-COSY correlation (bold line) observed for siphonodictyal E1 (**1**).

Siphonodictyal E1 (**1**) was identified by NMR data and its accurate mass of 598.1338 [M + Na − 2H]^−^ which indicated the molecular formula C_24_H_35_NO_11_S_2_. Comparison of the NMR data of **1** and siphonodictyal E (**9**, Figure 2) [2] revealed the presence of the identical sesquiterpene acyclic chain with one of the terminal methyl groups oxidized to a carboxylic acid. Moreover, the diagnostic values for C-14 (δ 15.7) and C-15 (δ 15.9) confirmed a common 6*E*, 10*E* double-bond geometry [6–7]. The NMR spectral data also suggested a similar aromatic ring system as in siphonodictyal E (**9**). Moreover, the ^1^H,^1^H-COSY correlation between the two methylene groups CH_2_-23 and CH_2_-24 as well as the ^1^H,^13^C-HMBC correlations from H-22 (δ 8.67) to C-18 (δ 144.0), C-19 (δ 156.4), C-20 (δ 110.9), C-21 (δ 141.8), and C-23 (δ 51.2) further indicated that an iminoethanesulfonic acid is connected to the aromatic ring system in **1** (Figure 3), as in siphonodictyal B1 (**6**, Figure 2) [5].

**Figure 4:**
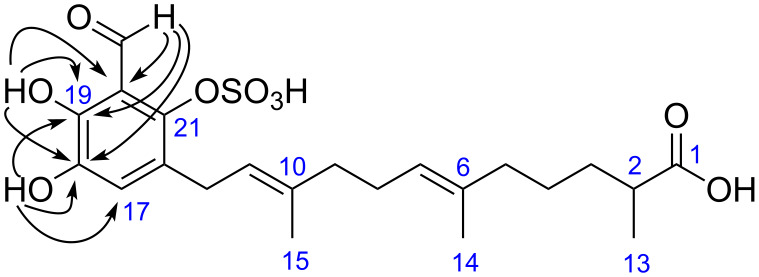
Selected ^1^H,^13^C-HMBC correlations (H → C) observed for siphonodictyal E2 (**2**).

**Figure 5:**
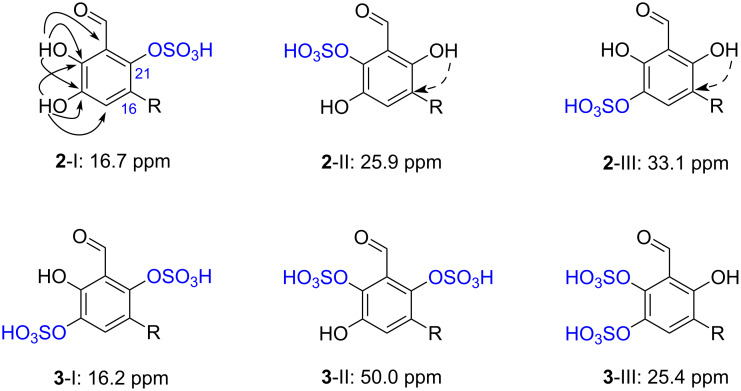
Possible constitutions for the aromatic moieties of siphonodictyals E2 (**2**) and E3 (**3**) (sum over all Δδ(^13^C): **2**-I 16.7 ppm, **2**-II 25.9 ppm, **2**-III 33.1 ppm, **3**-I 16.2 ppm, **3**-II 50.0 ppm, **3**-III 25.4 ppm); plain arrows in **2**-I showed the HMBC correlations from 18-OH and 19-OH to the aromatic carbons; dashed arrows in **2**-II and **2**-III indicate HMBC correlations which would be expected.

Examination of the NMR data for siphonodictyal E2 (**2**) revealed a high degree of similarity with the data reported for siphonodictyal E (**9**). However, the molecular ion peak of compound **2** was shown at *m*/*z* 469.1534 ([M − H]^−^ C_22_H_29_O_9_S), which has 80 amu more than that of **9**, indicating an additional sulfate ester group in compound **2**. The ^1^H,^13^C-HMBC correlations from 18-OH to C-17 (δ 122.8), C-18 (δ 141.8), and C-19 (δ 147.8) as well as from 19-OH to C-18 (δ 141.8), C-19 (δ 147.8), and C-20 (δ 115.5) indicated that C-18 and C-19 are connected to hydroxy groups rather than sulfate ester groups (Figure 4). Thus, the sulfate ester group should be at position C-21. Furthermore, the ^13^C NMR chemical shifts of the three constitutional isomers **2**-I to **2**-III were calculated using an increment system (Table 2) with additional values for sulfate esters published by Ragan [8]. These calculations (Table 3) allowed us to confirm the position of the sulfate moiety (**2**-I, Figure 5). The comparison of the NMR data of **2** and siphonodictyal B2 (**7**, Figure 2) [5] further confirmed the identity of the aromatic moiety of both compounds.

**Table 2:** ^13^C substituent effects Δδ in monosubstituted benzenes relative to the benzene carbon at 128.5 ppm [9].

Functional group	*i* [ppm]	*o* [ppm]	*m* [ppm]	*p* [ppm]

CHO	8.4	1.2	0.5	5.7
OH	26.9	−12.8	1.4	−7.4
Allyl	15.3	0	0.2	−2.4
OSO_3_H	22.0	−7.0	1.5	−2.0

**Table 3:** Results of the increment calculations for the aromatic moiety of the three constitutional proposals of siphonodictyal E2 (**2**) and siphonodictyal E3 (**3**).

Carbon No.	Siphonodictyal E2 (**2**)	**2**-I	**2**-II	**2**-III

16	125.9	131.3 (5.4)	130.9 (5.0)	125.6 (0.3)
17	122.8	124.3 (1.5)	124.3 (1.5)	130.0 (7.2)
18	141.8	141.3 (0.5)	141.7 (0.1)	131.0 (10.8)
19	147.8	142.9 (4.9)	137.9 (9.9)	148.6 (0.8)
20	115.5	118.7 (3.2)	118.7 (3.2)	113.0 (2.5)
21	144.5	145.7 (1.2)	150.7 (6.2)	156.0 (11.5)
Σ [ppm]		16.7	25.9	33.1

Carbon No.	Siphonodictyal E3 (**3**)	**3**-I	**3**-II	**3**-III

16	125.5	131.4 (5.9)	136.7 (11.2)	131.0 (5.5)
17	129.5	130.1 (0.6)	124.4 (5.1)	130.1 (0.6)
18	137.7	136.4 (1.3)	147.1 (9.4)	136.8 (0.9)
19	151.3	148.7 (2.6)	138.0 (13.3)	143.7 (7.6)
20	115.9	118.8 (2.9)	124.5 (8.6)	118.8 (2.9)
21	148.2	151.1 (2.9)	145.8 (2.4)	156.1 (7.9)
Σ [ppm]		16.2	50.0	25.4

The HRMS-ESI(−) spectrum for siphonodictyal E3 (**3**) displayed the pseudomolecular ion peak [M + Na − 2H]^−^ at *m*/*z* 539.1046, indicating the molecular formula C_22_H_30_O_10_S_2_. The ^13^C NMR spectrum contained six olefinic signals in addition to the aromatic signals, and the ^1^H NMR spectrum contained four olefinic methyl signals at δ 1.62 (s), δ 1.54 (s), δ 1.55 (s), and δ 1.66 (s), which suggested a sesquiterpene side chain containing three trisubstituted olefinic bonds. The two sulfate ester groups were assigned to attach to C-18 and C-21 after comparison of the NMR data of the aromatic moieties of **3** with those of siphonodictyal B3 (**8**, Figure 2). Nevertheless, the results of the increment calculations using the values for sulfate esters from Ragan [8] clearly favours the isomer **3**-I (Figure 5), which further supports our proposed structure.

**Figure 6:**
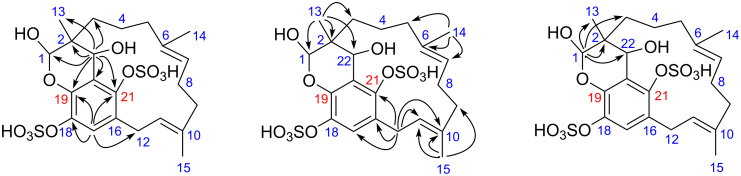
Selected ^1^H,^13^C-HMBC correlations (H → C) observed for siphonodictyal E4 (**4a**).

**Figure 7:**
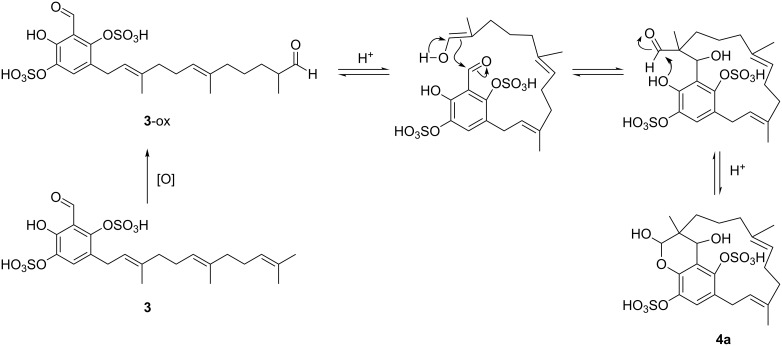
Proposed biogenesis of **4a** starting from the hypothetical precursor **3**-ox with an acyclic sesquiterpenoid part.

The HRMS-ESI(−) spectrum for siphonodictyal E4 (**4**) showed the pseudomolecular ion peak [M + Na − 2H]^−^ at *m*/*z* 555.0954, confirmed its molecular formula as C_22_H_30_O_11_S_2_, corresponding to eight double bond equivalents. The sesquiterpenoid moiety of the molecule contained two trisubstituted olefinic bonds (δ 121.7, δ 125.7, δ 131.9, δ 134.9) and a hemiacetal carbon (δ 96.2) at one terminus which differed from the acyclic side chain in **1** and **2**. The remaining six carbon signals in the downfield region of the ^13^C NMR spectrum belonged to the tetrasubstituted aromatic ring system. In contrast to **1** and **2**, compound **4** contained an oxymethine group (CH-22, δ_C_ 65.3, δ_H_ 4.44) which connected the sesquiterpene chain with the aromatic ring. This is supported by the six/seven HMBC correlations from H-22 to C-1 (δ 96.2), C-2 (δ 37.3), C-3 (δ 31.4), C-13 (δ 19.9), C-19 (δ 143.2) / C-21 (δ 142.9), and C-20 (δ 120.3). The HMBC correlations from H-12 (δ 2.58, 3.99) to C-17 (δ 120.8) and C-21 and from H-17 (δ 7.19) to C-18 (δ 137.2) confirmed the position of the unsubstituted CH-17 as shown in the proposed structure (Figure 6). Since the very weak HMBC correlation from H-1 to C-19 or C-21 could not be unambiguously assigned, the constitutional isomers with respect to the position of the oxygen bridge **4a** and **4b** (Figure 1) are in accordance with the NMR data. This is the first compound isolated from this sponge which contains a macrocycle. Moreover, this macrocycle could probably be formed by an aldol addition from a precursor with an acyclic sesquiterpene side chain (Figure 7). The aromatic ring system of proposed precursor **3**-ox in Figure 7 also appeared in the known compound siphonodictyal B3 (**8**) [5] isolated from the same sponge source as well as the newly identified compound **3**. There are no similar compounds known which contained the aromatic ring system with two adjacent sulfate ester groups. Therefore, we assumed that the constitutional isomer **4a** is more likely. The δ values of methyl carbons C-14 and C-15 below 20 ppm indicated the *E* configuration of the double bonds C-6/C-7 and C-10/C-11 of the isoprenoid chain [6–7]. Additionally, in the NOESY spectrum we could observe a weak correlation between H-22 (δ 4.44) and H-3 (δ 1.03) as well as between H-22 (δ 4.44) and H-13 (δ 1.04). Because of the overlapping of the proton signals of H-3 and H-13, this assignment is not unambiguous. Due to the flexibility of the macrocycle of **4** it was not possible to analyse the conformation of the pyrane. Therefore, the configurations at the centers C-1, C-2, and C-22 could not be assigned. Further investigations are hindered by the fact that compound **4** easily degrades.

The HRMS-ESI(−) spectrum for cyclosiphonodictyol A (**5**) showed the pseudomolecular ion peak [M + Na − 2H]^−^ at *m*/*z* 343.2292, confirmed its molecular formula as C_22_H_32_O_3_. The ^13^C NMR spectrum displayed six signals in the area of aromatic carbon atoms and 16 signals in the aliphatic region. The ^1^H,^13^C-HSQC spectrum indicated four methyl and seven methylene groups. Two of these methylene groups connected directly to the aromatic ring system indicated by the ^1^H,^13^C-HMBC correlations H-15 (δ 2.33, 3.06) → C-16 (δ 129.9), C-17(δ 146.4), and C-21 (δ 127.7) as well as H-22 (δ 4.50, 4.64) → C-16, C-20 (δ 146.5), and C-21. The sesquiterpene unit of **5** was identified by ^1^H,^1^H-COSY and ^1^H,^13^C-HMBC experiments. Furthermore, the HMBC correlations H-15 → C-8 (δ 78.5), C-9 (δ 58.1), and C-10 (δ 38.2) confirmed the connection between C-15 and C-9 and the HMBC correlation H-22 → C-8 indicated the existence of the oxygen bridge between C-22 and C-8. The *p*-hydroquinone moiety was established on the basis of the ^1^H and ^13^C NMR chemical shifts as well as the HMBC correlations mentioned above, namely, H-15 → C-16, C-17, C-21; H-22 → C-16, C-20, C-21. The relative configuration was confirmed by a ROESY experiment. The observed ROESY correlations between H-12 and H-13, H-13 and H-15, H-14 and H-15 suggested that the three methyl groups as well as CH_2_-15 are axial. Examination of the NMR data for **5** revealed a high degree of similarity with data reported for bis(sulfato)cyclosiphonodictyol A [10]. The difference concerned the absence of the two sulfate ester groups. Compound **5** could be the precursor of bis(sulfato)cyclosiphonodictyol A. Another possibility is that bis(sulfato)cyclosiphonodictyol A lost its sulfate ester group during the isolation procedure.

The new compounds were tested for antimicrobial activity against different Gram-positive and Gram-negative bacteria, fungi, and for antiproliferative activity. The results given in Table 4 show that siphonodictyal E3 (**3**) and cyclosiphonodictyol A (**5**) exhibited mild activity against the Gram-positive bacteria *Staphylococcus aureus* and *Micrococcus luteus*, while siphonodictyal E4 (**4**) show cytotoxic activity against the L929 mouse fibroblasts, KB-31 epidermoid carcinoma, and the breast cancer cell line MCF7, although none of them showed activity in the antimicrobial assays against Gram-negative bacteria and the fungus *Candida albicans*.

**Table 4:** Results of the antimicrobial and antiproliferation assays of compounds **1**–**5**. Minimum inhibiting concentrations (MIC) for the antimicrobial assays and the IC_50_ values for the antiproliferation assays are given in µM.

		**1**	**2**	**3**	**4**	**5**

Gram-negative bacteria	*Pseudomonas aeruginosa*	–	–	–	–	–
	*Klebsiella pneumonia*	–	–	–	–	–
Gram-positive bacteria	*Staphylococcus aureus* (MRSA)	–	–	37	–	117
	*Staphylococcus aureus* (MSSA)	–	–	74	–	117
	*Micrococcus luteus*	–	–	74	–	58
Fungi	*Candida albicans*	–	–	–	–	–
Cytotoxic activity	L929 mouse fibroblasts	–	–	–	27	–
	KB31 epidermoid carcinoma	–	–	–	108	–
	MCF7 breast cancer	–	–	–	54	175
	FS4-LTM conditional immortalization human fibroblasts	–	–	–	–	–

**Figure 8:**
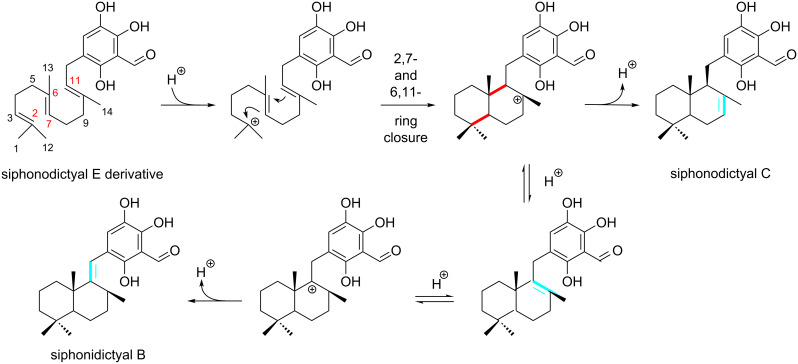
Hypothetical biogenesis of the bicyclic sesquiterpenoid moiety from the acyclic precursor of the siphonodictyals in the sponge *Aka coralliphagum* [11]. The newly formed bonds from the acyclic to the bicyclic sesquiterpenoid moiety are drawn in red. The isomerizing double bond is drawn in blue.

Many of the isolated compounds from *Aka coralliphagum* are bicyclic sesquiterpene hydroquinones. However, in the course of our investigation, three linear sesquiterpenes were isolated. From a biosynthetical point of view [11], we propose that the linear sesquiterpenes are the precursors of the bicyclic sesquiterpenes (Figure 8). Therefore, siphonodictyals B1–B3 (**6**–**8**) could be derived from siphonodictyals E1–E3 (**1**–**3**), respectively.

Aromatic sulfates occur in a wide range of higher plants and animals, particularly those growing in marine, estuarine, or freshwater environments. This is probably due to the high content of inorganic sulfate in aquatic environments (both limnic and marine environments – seawater contains an average of 28.7 mM inorganic sulfate [12]). Jensen and Ragan have demonstrated the presence of the natural product 1,2,3,5-tetrahydroxybenzene-2,5-disulfate in the marine brown alga *Ascophyllum nodosum* [13]. Kobayashi et al. isolated halenaquinol sulfate from the marine sponge *Xestospongia sapra* [14].

In the course of our investigation on the sponge *Aka coralliphagum*, we isolated a number of compounds containing sulfated phenols. These sulfated metabolites are labile [5], and easily loose the sulfate ester groups by hydrolysis in water [15]. Moreover, the influence of the sulfate esters on the bioactivity has been proven by activity tests: the desulfated compounds tend to be more active [5]. Therefore, we propose that the sulfate esters act as hydroxy protecting groups. The sponges excrete the sesquiterpene hydroquinones primarily in the sulfated form and their activities get increased by hydrolysis of these labile groups. This would lead to a prolonged bioactivity of these metabolites and therefore to a more efficient defense against predators or pathogens.

## Experimental

^1^H and ^13^C NMR spectra were recorded on a Bruker Avance 600 MHz NMR spectrometer equipped with a cryo platform and a Bruker Avance 400 MHz NMR spectrometer. All experiments were measured at 303 K. HPLC separation was achieved by a Kromasil RP-18 column (16 mm × 250 mm, 5 µm) applying a H_2_O (5 mM NH_4_OAc)/MeCN/MeOH gradient. UV spectra were recorded during HPLC separation with a DAD detector (Jasco MD-2010 Plus). Accurate mass spectra were acquired using a Bruker micrOTOF_LC_ mass spectrometer equipped with an ESI source. Mass calibration was performed using a sodium formate cluster prior each experiment.

The sponges were collected by SCUBA diving at a depth of 29 m off the coast of San Salvador in the Bahamas in June 2008. The samples were immediately frozen after collection and kept at −20 °C until extraction. A voucher specimen of this species is deposited in our lab (voucher number: *AKA* 08/45). The freeze-dried sponge sample (107.6 g) was extracted three times at room temperature with DCM/MeOH (1:1). After filtration, the filtrate was evaporated to yield 35.5 g crude extract which was further partitioned between *n*-hexane and methanol. The methanol extract was dried and followed with the partitioning between *n*-BuOH and H_2_O. The resulting *n*-BuOH phase was concentrated (9.2 g) and purified by liquid chromatography using RP-18 silica gel with a stepwise gradient from H_2_O/MeOH (100:0), (85:15), (50:50) to (0:100) yielding fractions A, B, C, and D, respectively. The fraction B was further purified by HPLC using an RP-18 column yielding compounds **1** (2.2 mg, HPLC gradient flow: H_2_O (5 mM NH_4_OAc)/MeCN: 0 min 81:19, 40 min 75:25), **2** (3.1 mg, HPLC isocratic flow: H_2_O (5 mM NH_4_OAc)/MeCN: 87:13 40 min), **4** (1.3 mg, HPLC gradient flow: H_2_O (5 mM NH_4_OAc)/MeCN: 0 min 85:15, 40 min 83:17, 60 min 80:20), and **5** (3.0 mg, HPLC gradient flow: H_2_O (5 mM NH_4_OAc)/MeCN: 0 min 85:15, 40 min 83:17, 60 min 80:20). The fraction C was also further purified by HPLC using the same RP-18 column yielding **3** (2.5 mg, HPLC gradient flow: H_2_O (5 mM NH_4_OAc)/MeCN: 0 min 76:24, 40 min 74:26, 45 min 70:30)

## Antimicrobial assay

Determination of antimicrobial activities was carried out by the Helmholtz Center for Infection Research (HZI), and was based on the microdilution test, using 96-well micro titration plates. The MIC was defined as 50% growth inhibition after 24 hour incubation compared to that in the growth control well.

### Cytotoxic assay

The cytotoxicity was determined using WST-1 assays, targeting cell lines L929 mouse fibroblasts, KB-31 epidermoid carcinoma, MCF-7 breast cancer, and FS4-LTM conditional immortalization human fibroblasts, respectively. The FS4-LTM cell line was incubated for 24 hours with the tested substances. The other cell lines were incubated for 5 days with the tested substances.

#### Experimental data

Siphonodictyal E1 (**1**): yellow powder; ^1^H NMR (DMSO-*d*_6_, 600 MHz) and ^13^C NMR (DMSO-*d*_6_, 150 MHz) see Table 1; HRMS-ESI(−) *m/z*: [M + Na − 2H]^−^ calcd for C_24_H_33_NO_11_S_2_Na, 598.1398; found, 598.1338.

Siphonodictyal E2 (**2**): yellow powder; ^1^H NMR (DMSO-*d*_6_, 600 MHz) and ^13^C NMR (DMSO-*d*_6_, 150 MHz) see Table 1; HRMS-ESI(−) *m/z*: [M − H]^−^ calcd for C_22_H_29_O_9_S, 469.1538; found, 469.1561.

Siphonodictyal E3 (**3**): yellow powder; ^1^H NMR (DMSO-*d*_6_, 600 MHz) and ^13^C NMR (DMSO-*d*_6_, 150 MHz) see Table 1; HRMS-ESI(−) *m/z*: [M + Na − 2H]^−^ calcd for C_22_H_28_O_10_S_2_Na, 539.1027; found, 539.1046.

Siphonodictyal E4 (**4**): yellow powder; CD data {(MeOH) 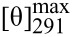 = +37}; ^1^H NMR (DMSO-*d*_6_, 600 MHz) and ^13^C NMR (DMSO-*d*_6_, 150 MHz) see Table 1; HRMS-ESI(−) *m/z*: [M + Na − 2H]^−^ calcd for C_22_H_28_O_11_S_2_Na, 555.0976; found, 555.0954.

Cyclosiphonodictyol A (**5**): colorless powder; CD data {(MeOH) 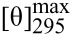 = +163}; ^1^H NMR (DMSO-*d*_6_, 600 MHz) and ^13^C NMR (DMSO-*d*_6_, 150 MHz) see Table 1; HRMS-ESI(−) *m/z*: [M − H]^−^ calcd for C_22_H_31_O_3_, 343.2279; found, 343.2292.

## Supporting Information

File 1 1D and 2D NMR spectra of the isolated compounds.
