# Supplementary material for: New sesquiterpene hydroquinones from the Caribbean sponge Aka coralliphagum
Source: Beilstein J Org Chem. 2014 Mar 6;10:613–21. doi: 10.3762/bjoc.10.52 (PMC3999825; doi:10.3762/bjoc.10.52)
Supplement: File 1 — 1D and 2D NMR spectra of the isolated compounds. [file Beilstein_J_Org_Chem-10-613-s001.pdf]

Supporting Information

for

New sesquiterpene hydroquinones from the Caribbean sponge *Aka coralliphagum*

Qun Göthel and Matthias Köck*

Address: Alfred-Wegener-Institut, Helmholtz-Zentrum für Polar- und Meeresforschung, Am Handelshafen 12, 27570 Bremerhaven, Germany

* Corresponding author

E-mail: Matthias Köck* - mkoeck@awi.de

1D and 2D NMR spectra of the isolated compounds

Figure S1: 1D ^{1}H NMR spectrum of siphonodictyal E1 (**1**) in DMSO-d_6, 303 K, 600 MHz................. S2
Figure S2: 1D ^{13}C NMR spectrum of siphonodictyal E1 (**1**) in DMSO-d_6, 303 K, 150 MHz................ S2
Figure S3: 2D ^{1}H,^{13}C-HSQC spectrum of siphonodictyal E1 (**1**) in DMSO-d_6, 303 K.......................... S3
Figure S4: 2D ^{1}H,^{13}C-HMBC spectrum of siphonodictyal E1 (**1**) in DMSO-d_6, 303 K......................... S3
Figure S5: 2D ^{1}H,^{1}H-COSY spectrum of siphonodictyal E1 (**1**) in DMSO-d_6, 303 K.......................... S4
Figure S6: 1D ^{1}H NMR spectrum of siphonodictyal E2 (**2**) in DMSO-d_6, 303 K, 600 MHz................. S5
Figure S7: 1D ^{13}C NMR spectrum of siphonodictyal E2 (**2**) in DMSO-d_6, 303 K, 150 MHz................ S5
Figure S8: 2D ^{1}H,^{13}C-HSQC spectrum of siphonodictyal E2 (**2**) in DMSO-d_6, 303 K.......................... S6
Figure S9: 2D ^{1}H,^{13}C-HMBC spectrum of siphonodictyal E2 (**2**) in DMSO-d_6, 303 K......................... S6
Figure S10: 2D ^{1}H,^{1}H-COSY spectrum of siphonodictyal E2 (**2**) in DMSO-d_6, 303 K......................... S7
Figure S11: 1D ^{1}H NMR spectrum of siphonodictyal E3 (**3**) in DMSO-d_6, 303 K, 600 MHz S8
Figure S12: 1D ^{13}C NMR spectrum of siphonodictyal E3 (**3**) in DMSO-d_6, 303 K, 150 MHz S8
Figure S13: 2D ^{1}H,^{13}C-HSQC spectrum of siphonodictyal E3 (**3**) in DMSO-d_6, 303 K S9
Figure S14: 2D ^{1}H,^{13}C-HMBC spectrum of siphonodictyal E3 (**3**) in DMSO-d_6, 303 K S9
Figure S15: 2D ^{1}H,^{1}H-COSY spectrum of siphonodictyal E3 (**3**) in DMSO-d_6, 303 K....................... S10
Figure S16: 1D ^{1}H NMR spectrum of siphonodictyal E4 (**4**) in DMSO-d_6, 303 K, 600 MHz S11
Figure S17: 1D ^{13}C NMR spectrum of siphonodictyal E4 (**4**) in DMSO-d_6, 303 K, 150 MHz S11
Figure S18: 2D ^{1}H,^{13}C-HSQC spectrum of siphonodictyal E4 (**4**) in DMSO-d_6, 303 K S12
Figure S19: 2D ^{1}H,^{13}C-HMBC spectrum of siphonodictyal E4 (**4**) in DMSO-d_6, 303 K S12
Figure S20: 2D ^{1}H,^{1}H-COSY spectrum of siphonodictyal E4 (**4**) in DMSO-d_6, 303 K....................... S13
Figure S21: 1D ^{1}H NMR spectrum of cyclosiphonodictyol A (**5**) in DMSO-d_6, 303 K, 600 MHz S14
Figure S22: 1D ^{13}C NMR spectrum of cyclosiphonodictyol A (**5**) in DMSO-d_6, 303 K, 150 MHz S14
Figure S23: 2D ^{1}H,^{13}C-HSQC spectrum of cyclosiphonodictyol A (**5**) in DMSO-d_6, 303 K S15
Figure S24: 2D ^{1}H,^{13}C-HMBC spectrum of cyclosiphonodictyol A (**5**) in DMSO-d_6, 303 K S15
Figure S25: 2D ^{1}H,^{1}H-COSY spectrum of cyclosiphonodictyol A (**5**) in DMSO-d_6, 303 K S16

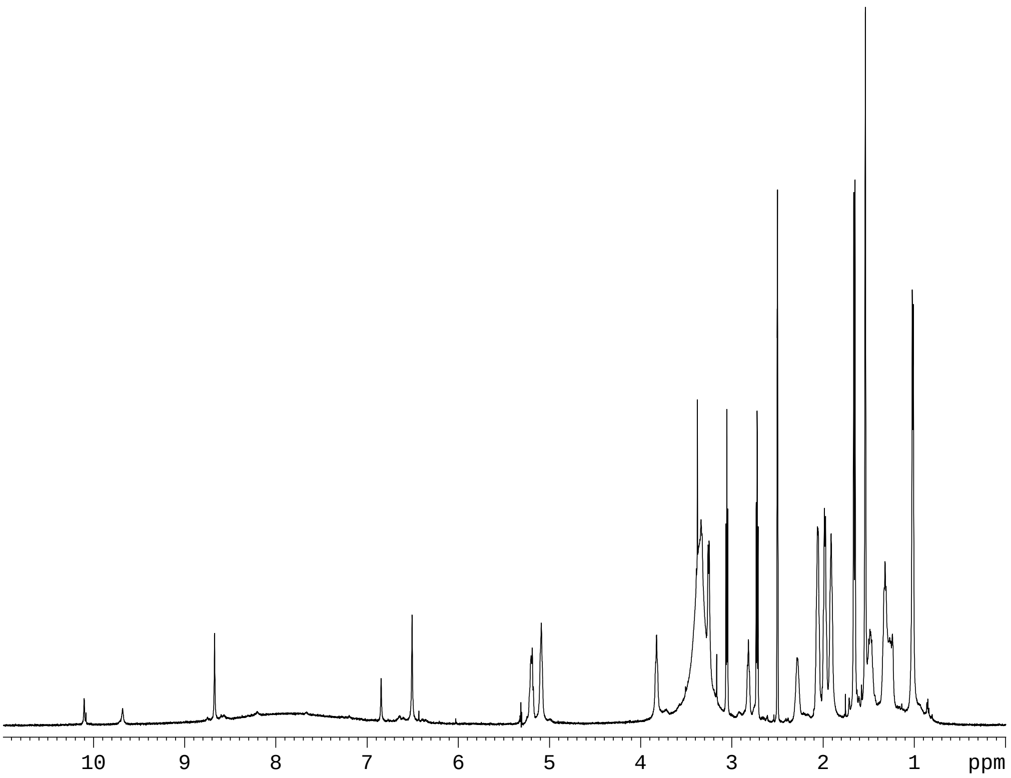

Figure S1: 1D ^{1}H NMR spectrum of siphonodictyal E1 (**1**) in DMSO-d_6, 303 K, 600 MHz.

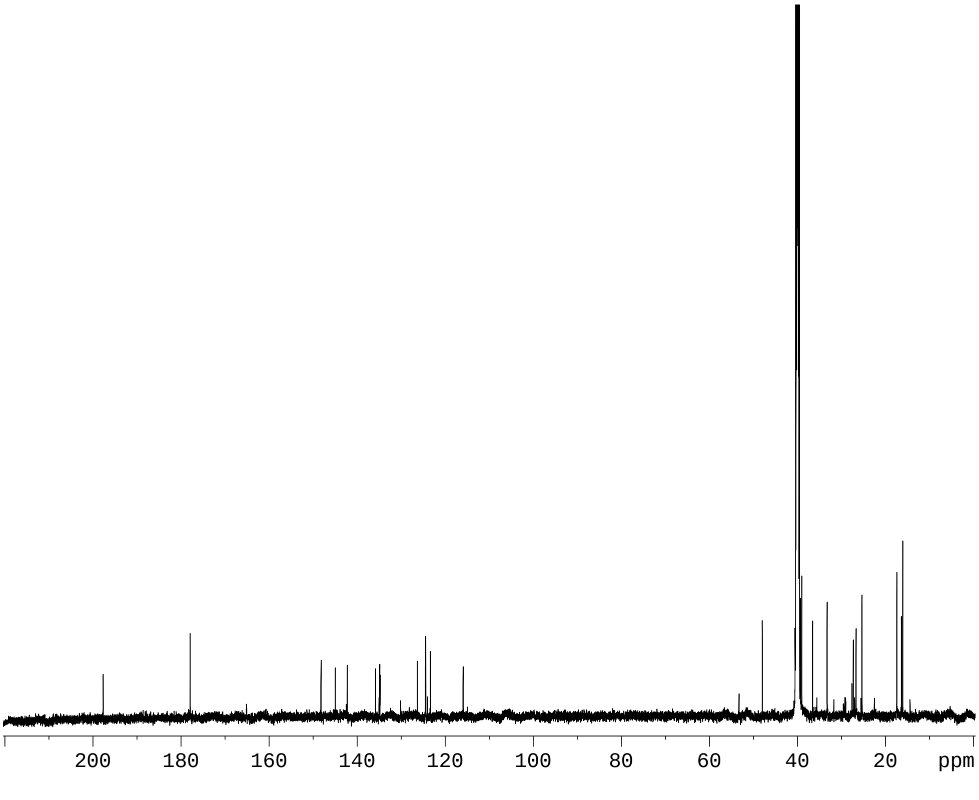

Figure S2: 1D ^{13}C NMR spectrum of siphonodictyal E1 (**1**) in DMSO-d_6, 303 K, 150 MHz.

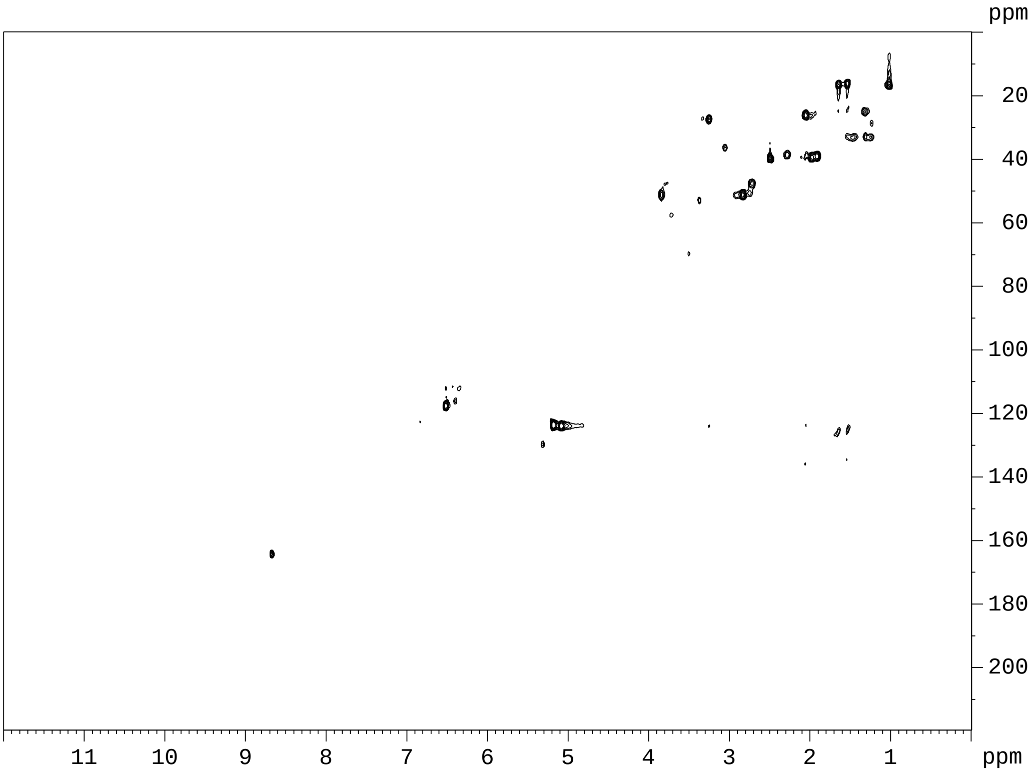

Figure S3: 2D ^{1}H,^{13}C-HSQC spectrum of siphonodictyal E1 (**1**) in DMSO-d_6, 303 K.

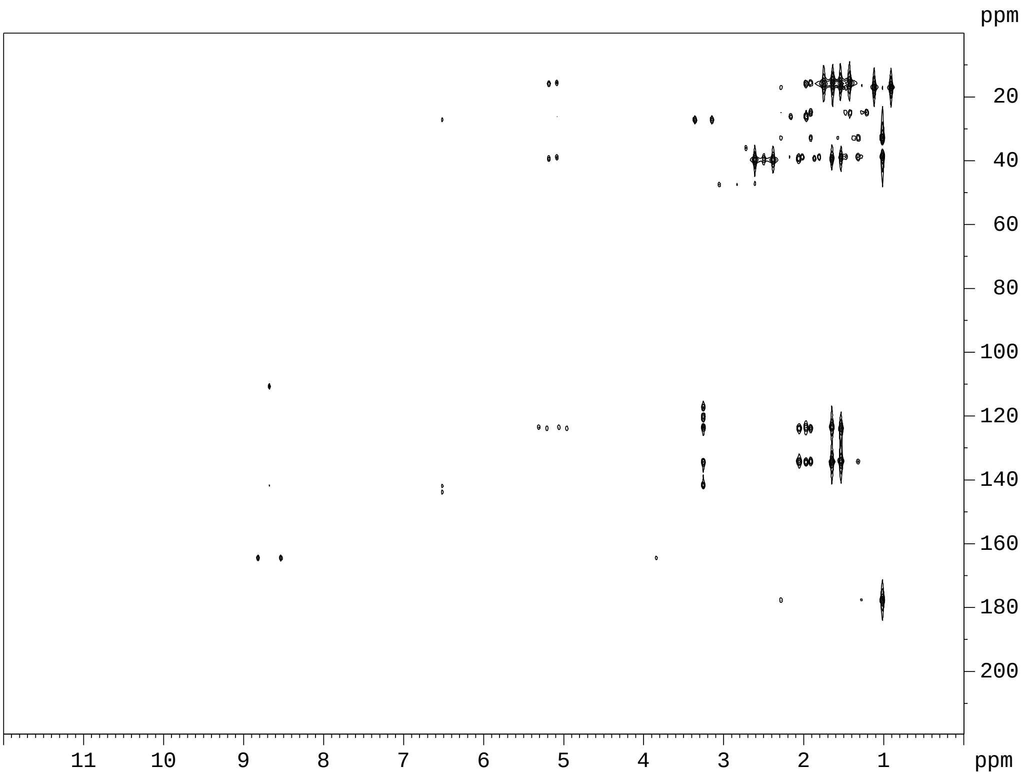

Figure S4: 2D ^{1}H,^{13}C-HMBC spectrum of siphonodictyal E1 (**1**) in DMSO-d_6, 303 K.

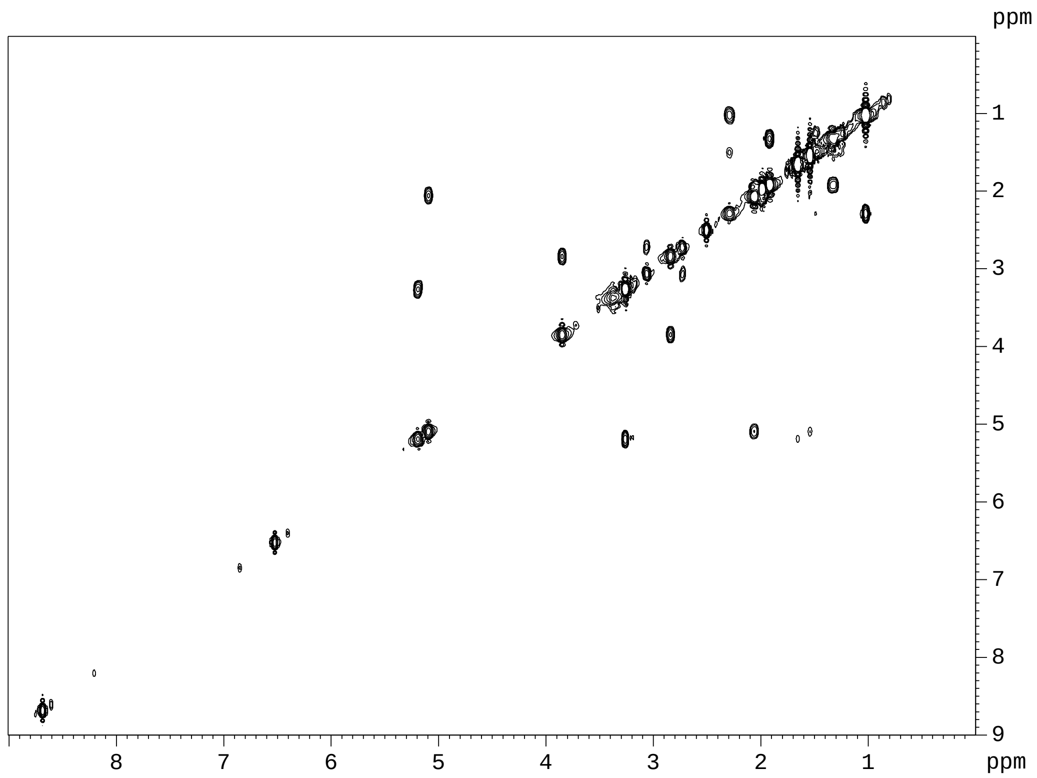

Figure S5: 2D ^{1}H,^{1}H-COSY spectrum of siphonodictyal E1 (**1**) in DMSO-d_6, 303 K.

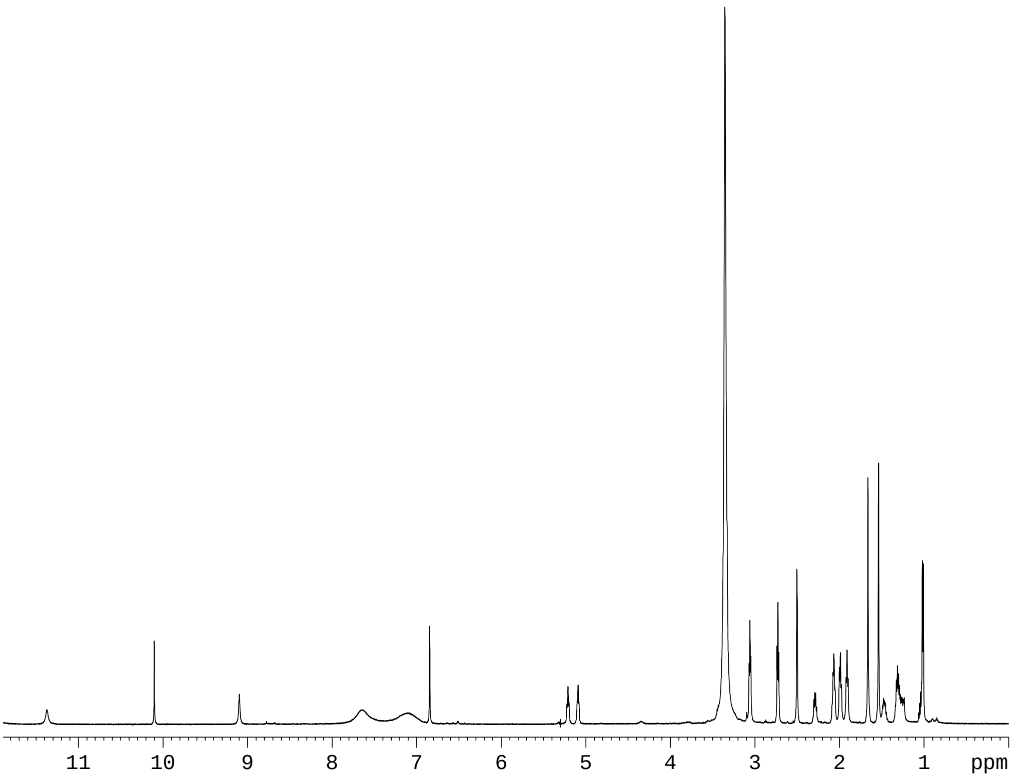

Figure S6: 1D ^{1}H NMR spectrum of siphonodictyal E2 (**2**) in DMSO-d_6, 303 K, 600 MHz.

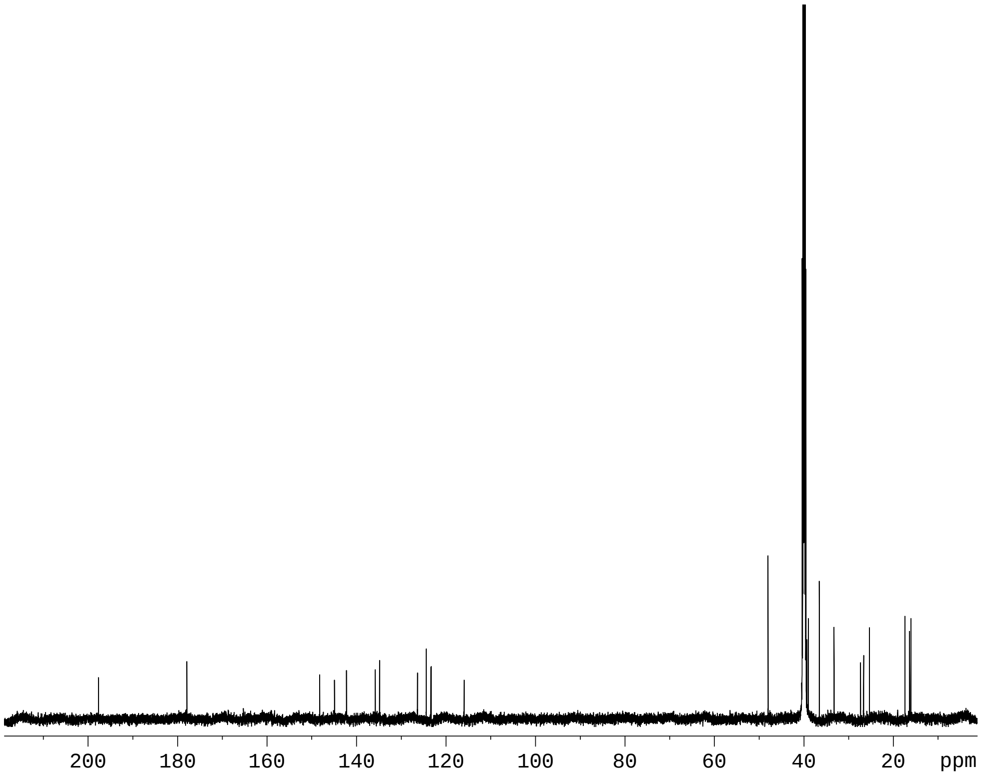

Figure S7: 1D ^{13}C NMR spectrum of siphonodictyal E2 (**2**) in DMSO-d_6, 303 K, 150 MHz.

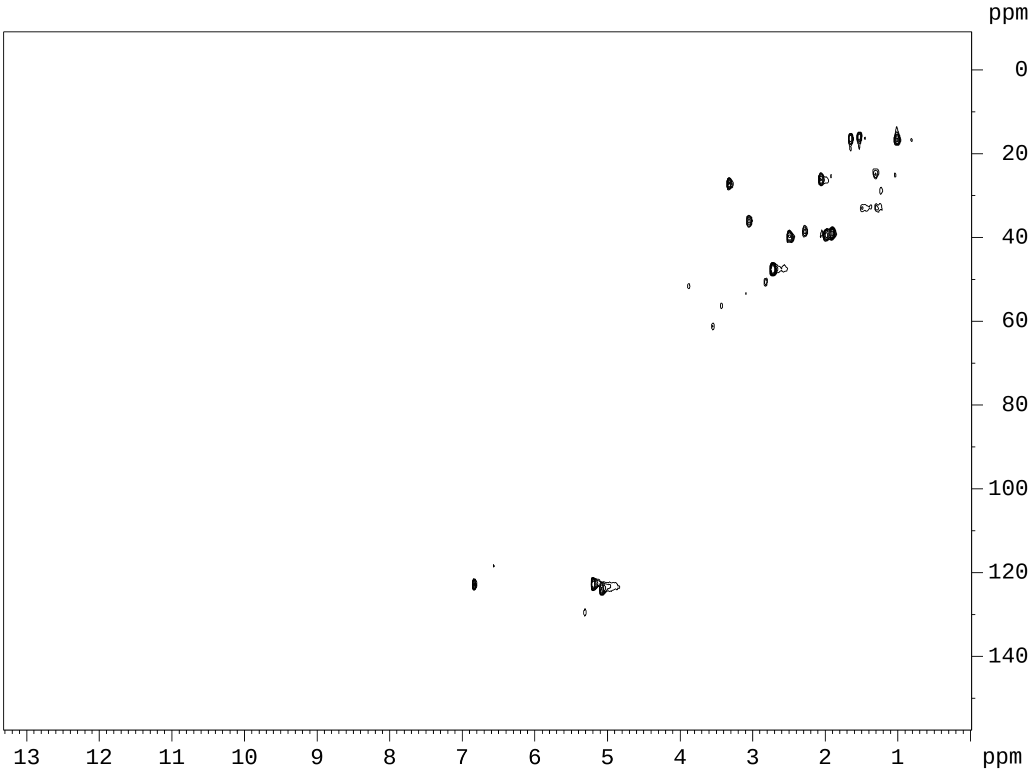

Figure S8: 2D $^{1}H,^{13}C$-HSQC spectrum of siphonodictyal E2 (**2**) in DMSO-d_6, 303 K.

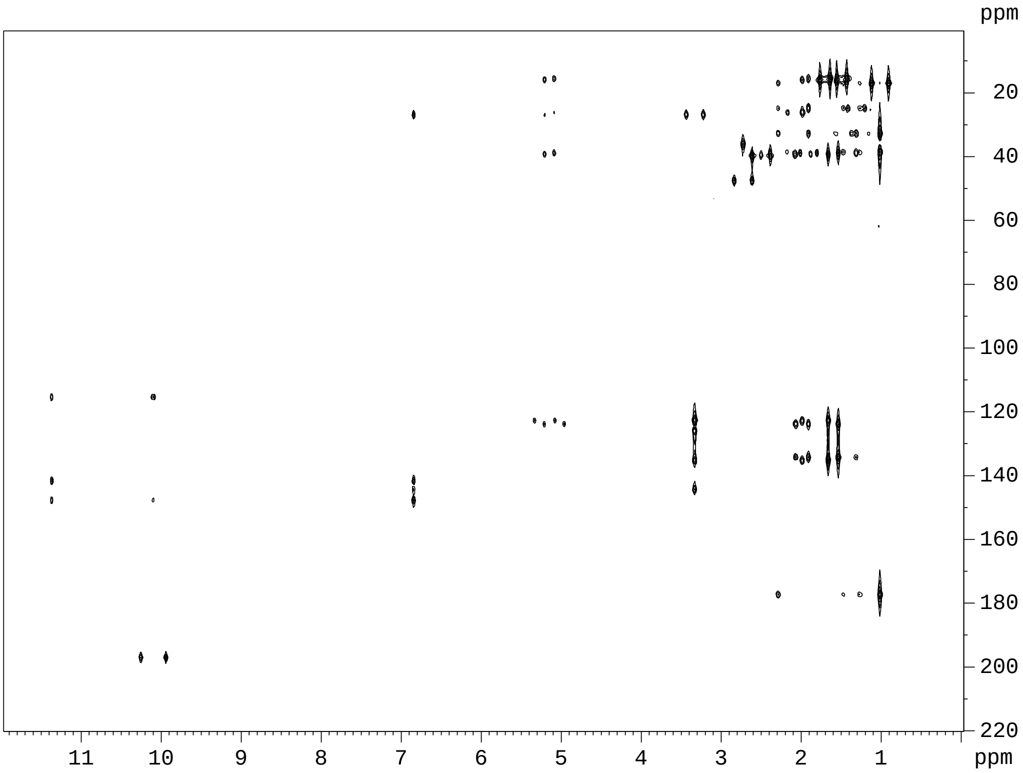

Figure S9: 2D $^{1}H,^{13}C$-HMBC spectrum of siphonodictyal E2 (**2**) in DMSO-d_6, 303 K.

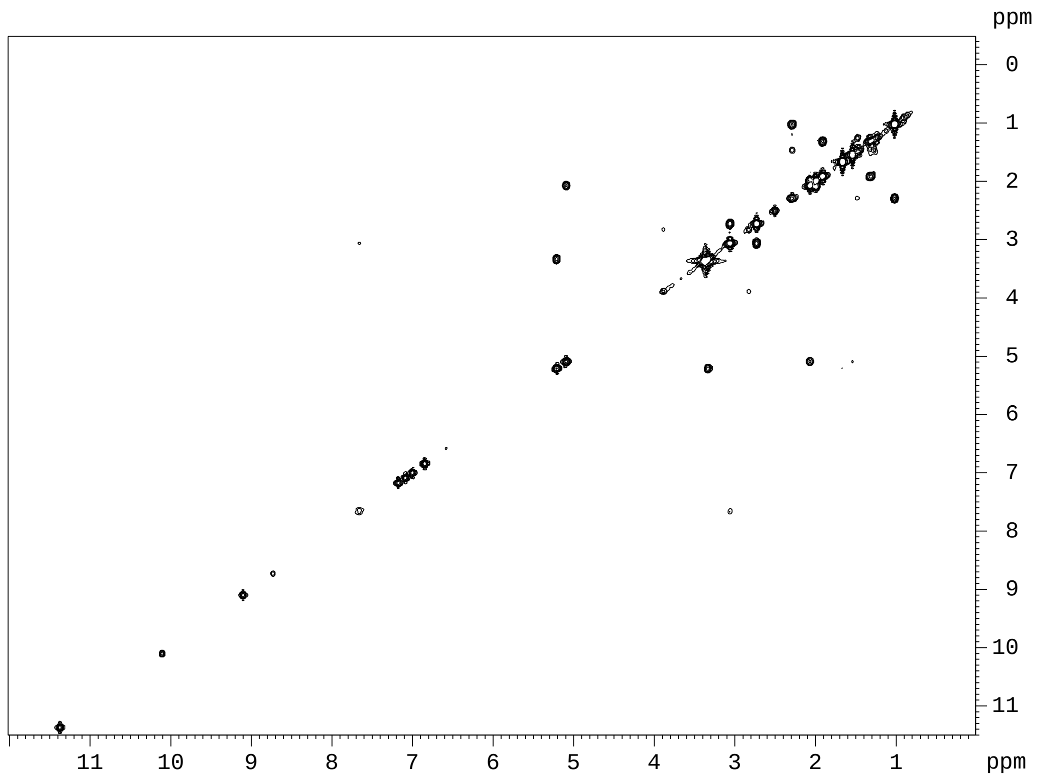

Figure S10: 2D ^{1}H,^{1}H-COSY spectrum of siphonodictyal E2 (**2**) in DMSO-d_6, 303 K.

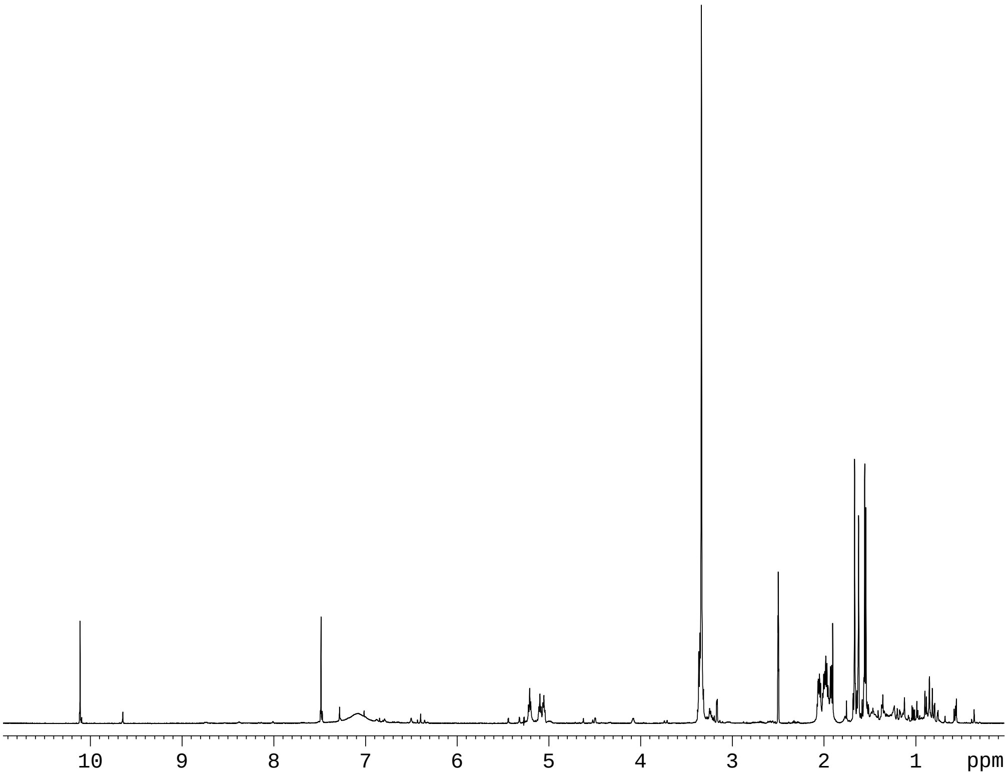

Figure S11: 1D ^{1}H NMR spectrum of siphonodictyal E3 (**3**) in DMSO-d_6, 303 K, 600 MHz.

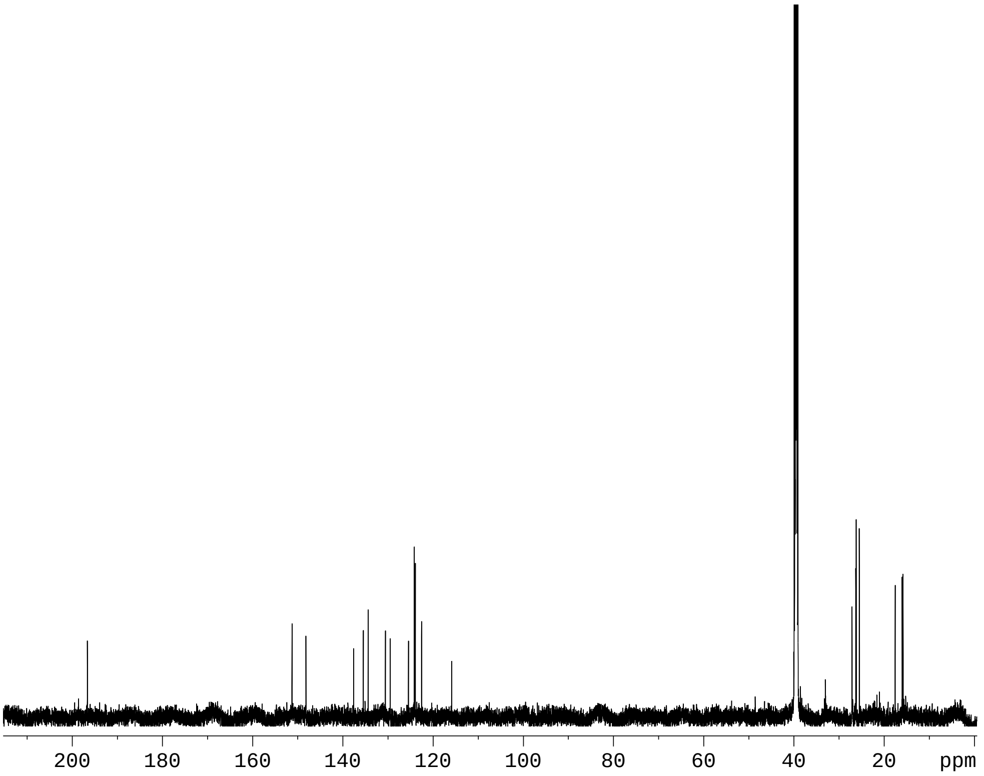

Figure S12: 1D ^{13}C NMR spectrum of siphonodictyal E3 (**3**) in DMSO-d_6, 303 K, 150 MHz.

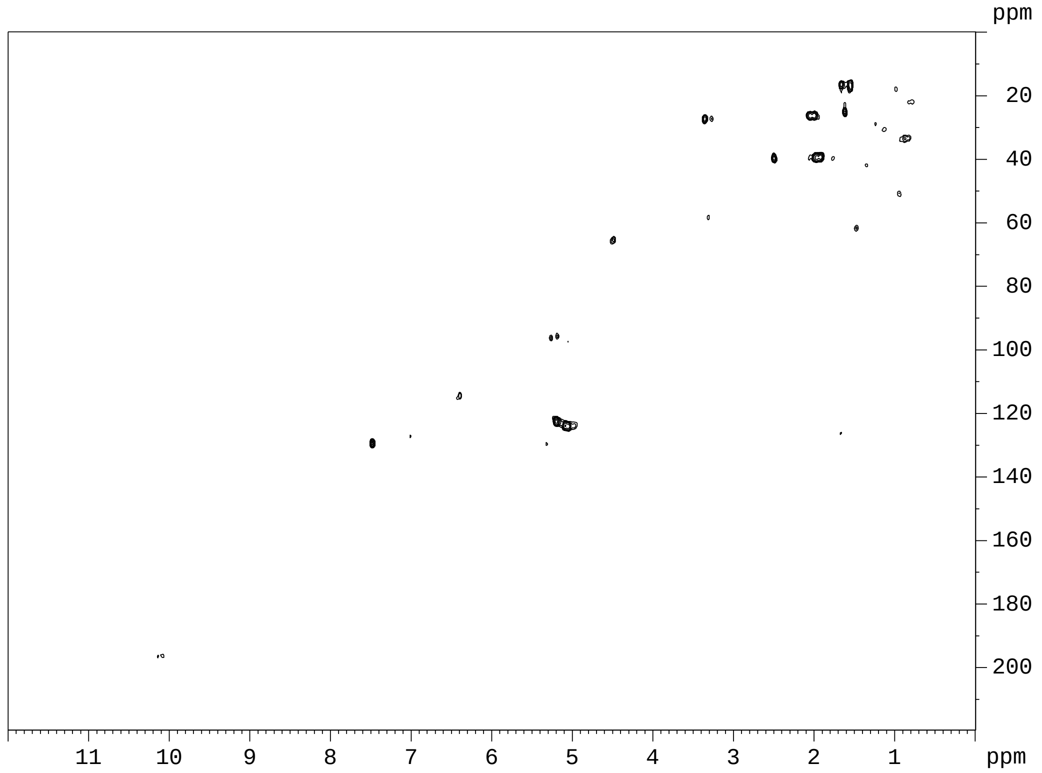

Figure S13: 2D $^{1}H,^{13}C$-HSQC spectrum of siphonodictyal E3 (**3**) in DMSO-d_6, 303 K.

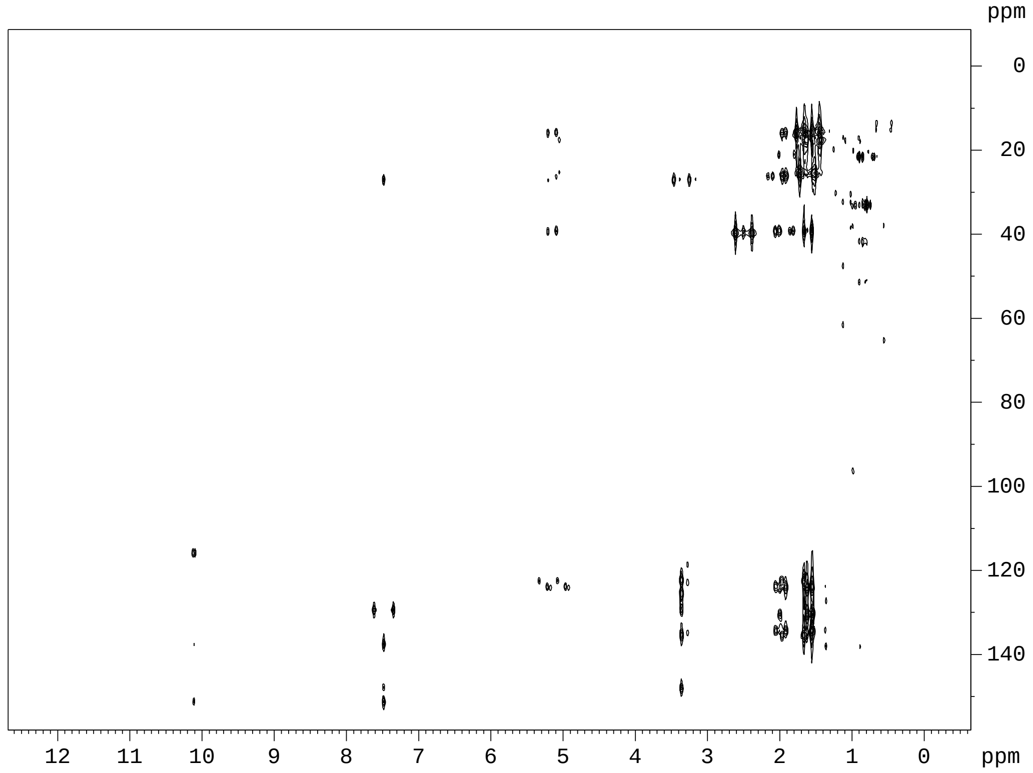

Figure S14: 2D $^{1}H,^{13}C$-HMBC spectrum of siphonodictyal E3 (**3**) in DMSO-d_6, 303 K.

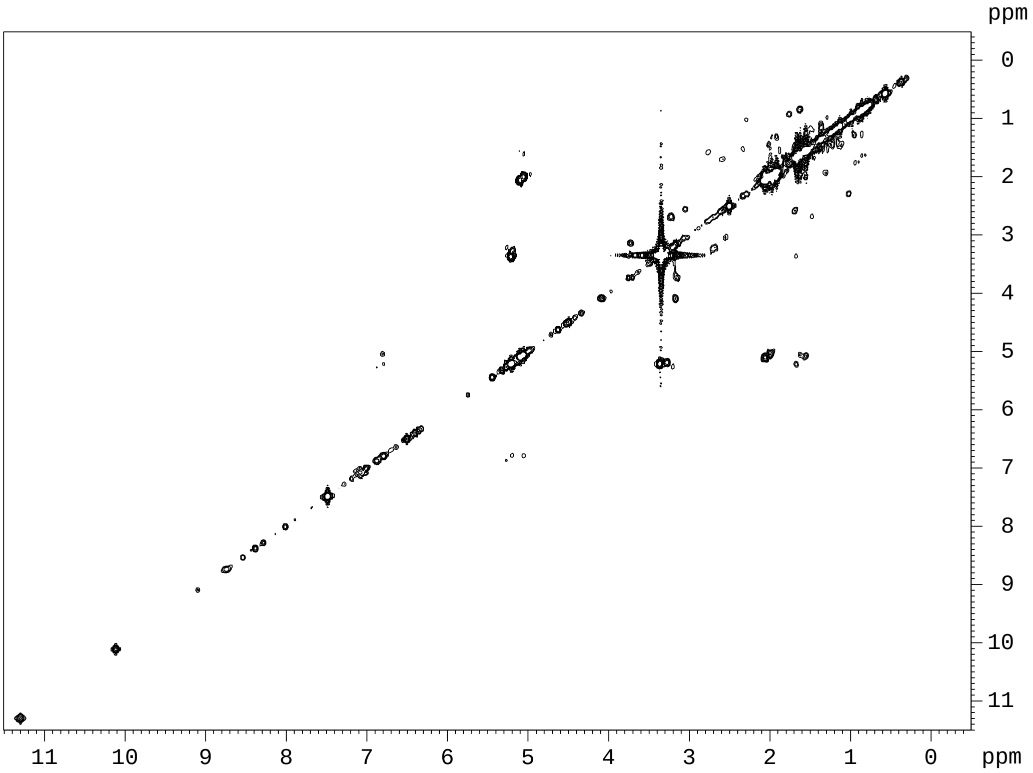

Figure S15: 2D ^{1}H,^{1}H-COSY spectrum of siphonodictyal E3 (**3**) in DMSO-d_6, 303 K.

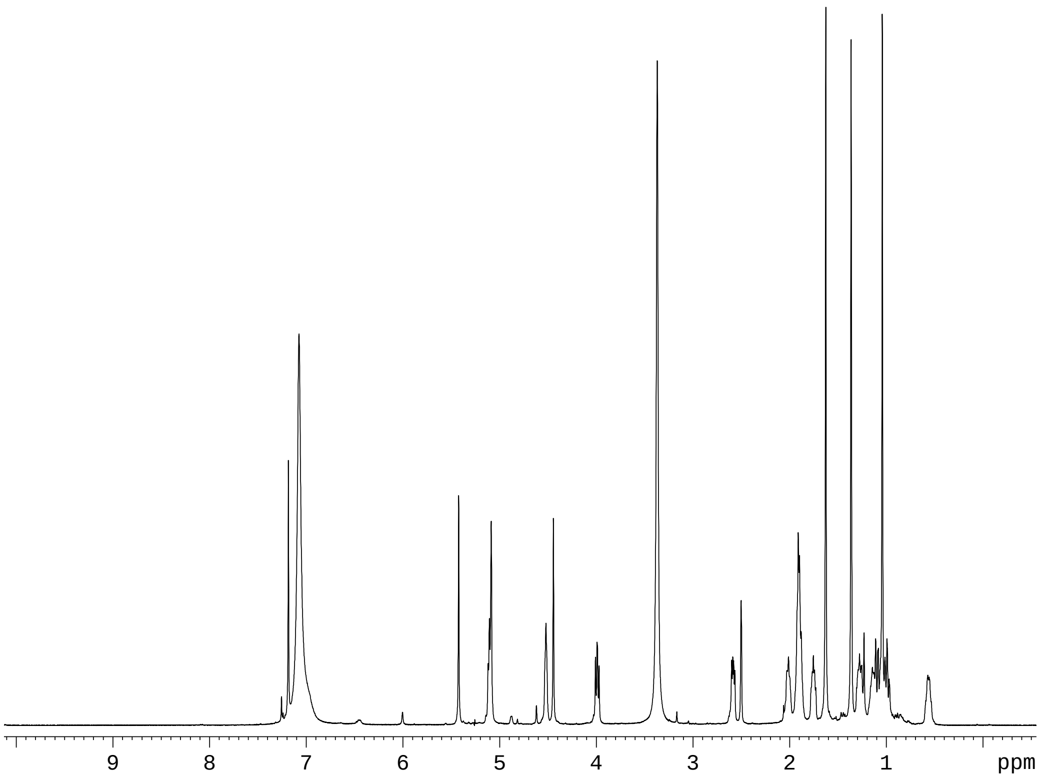

Figure S16: 1D ^{1}H NMR spectrum of siphonodictyal E4 (**4**) in DMSO-d_6, 303 K, 600 MHz.

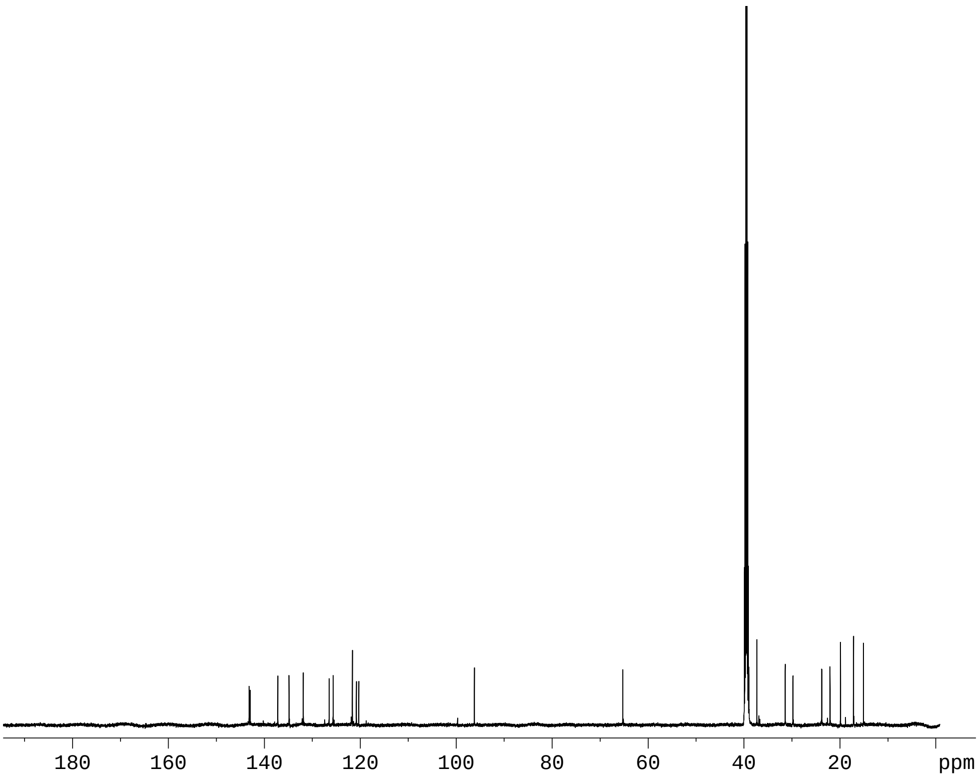

Figure S17: 1D ^{13}C NMR spectrum of siphonodictyal E4 (**4**) in DMSO-d_6, 303 K, 150 MHz.

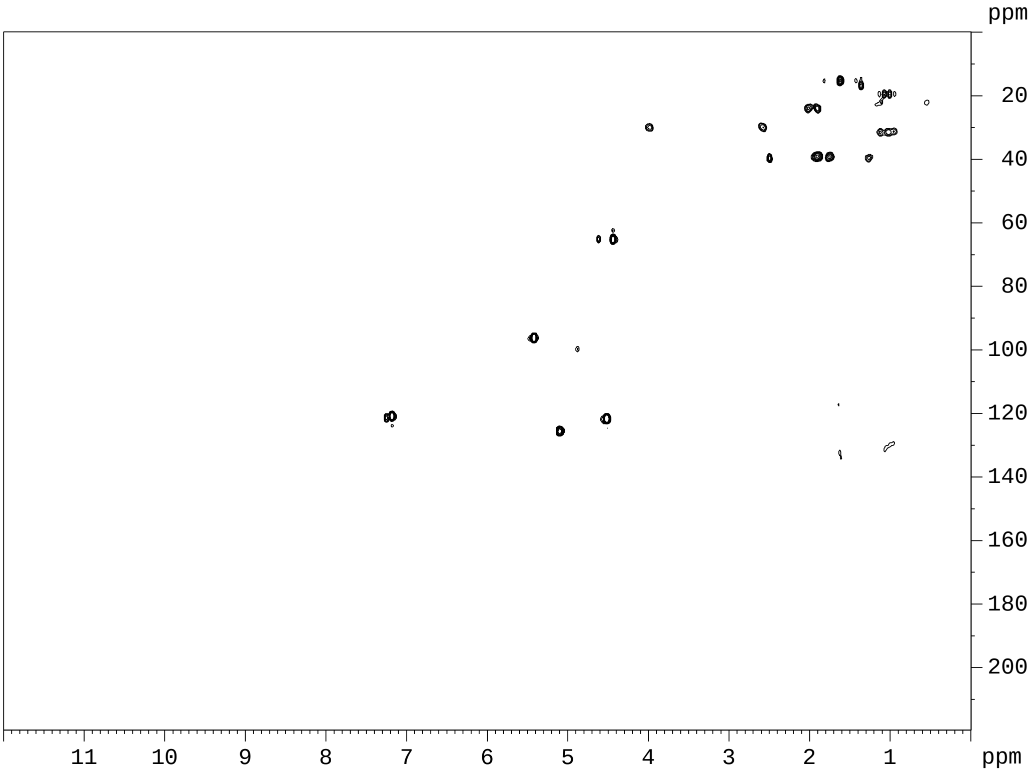

Figure S18: 2D ^{1}H,^{13}C-HSQC spectrum of siphonodictyal E4 (**4**) in DMSO-d_6, 303 K.

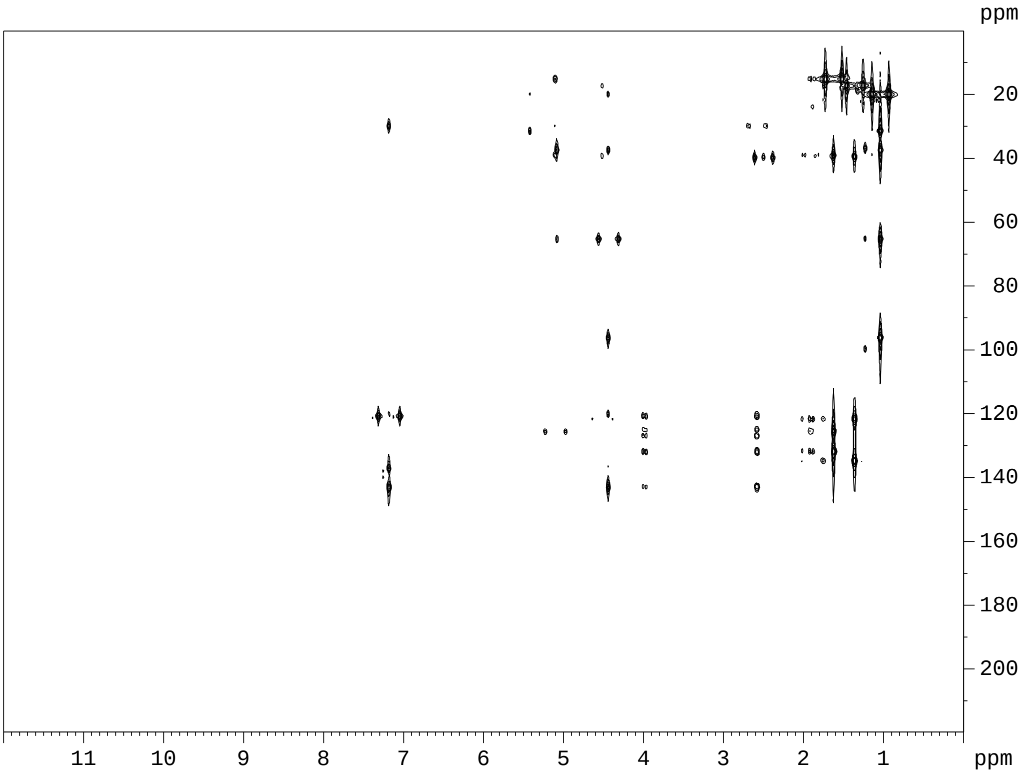

Figure S19: 2D ^{1}H,^{13}C-HMBC spectrum of siphonodictyal E4 (**4**) in DMSO-d_6, 303 K.

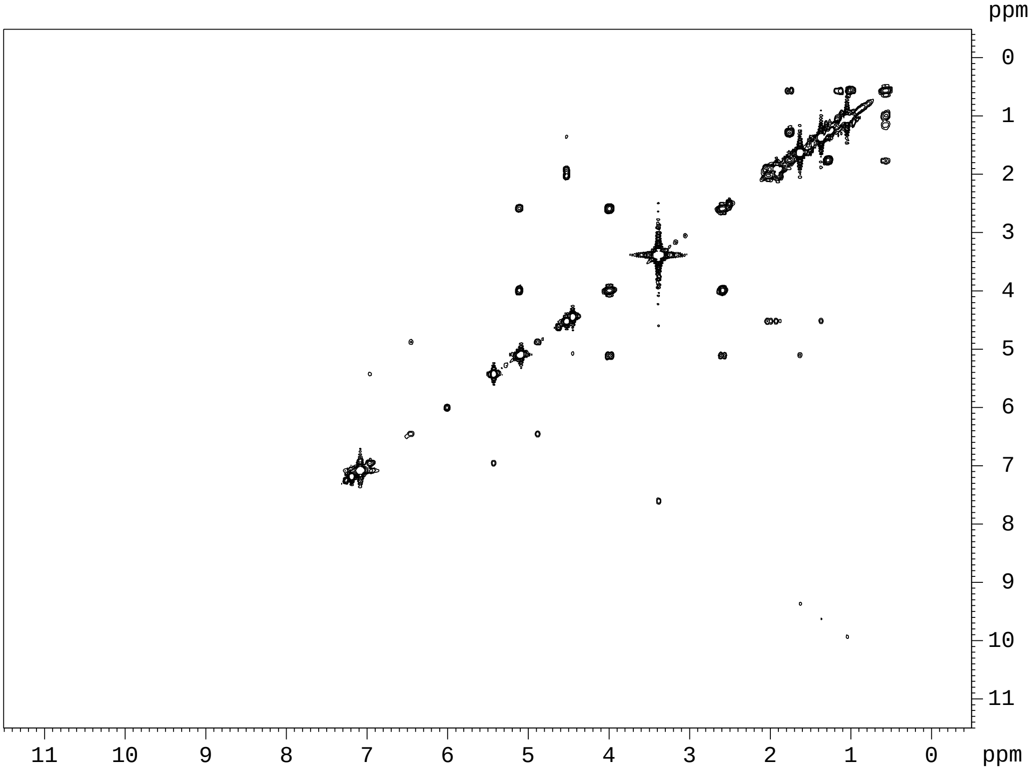

Figure S20: 2D ^{1}H,^{1}H-COSY spectrum of siphonodictyal E4 (**4**) in DMSO-d_6, 303 K.

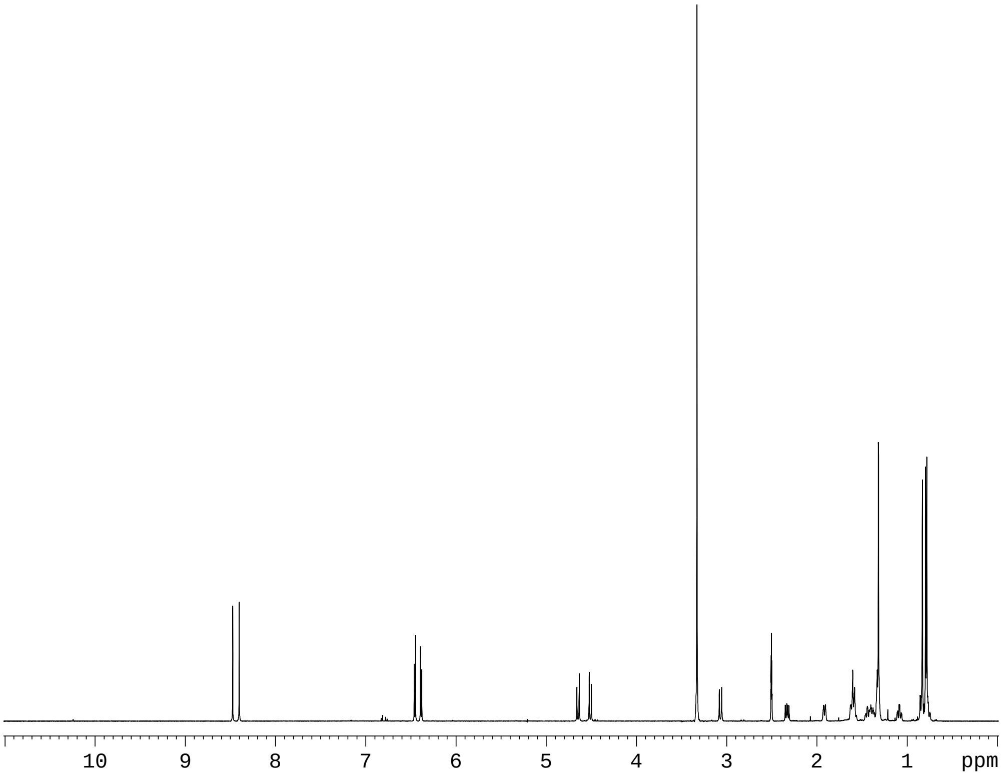

Figure S21: 1D ^{1}H NMR spectrum of cyclosiphonodictyol A (**5**) in DMSO-d_6, 303 K, 600 MHz.

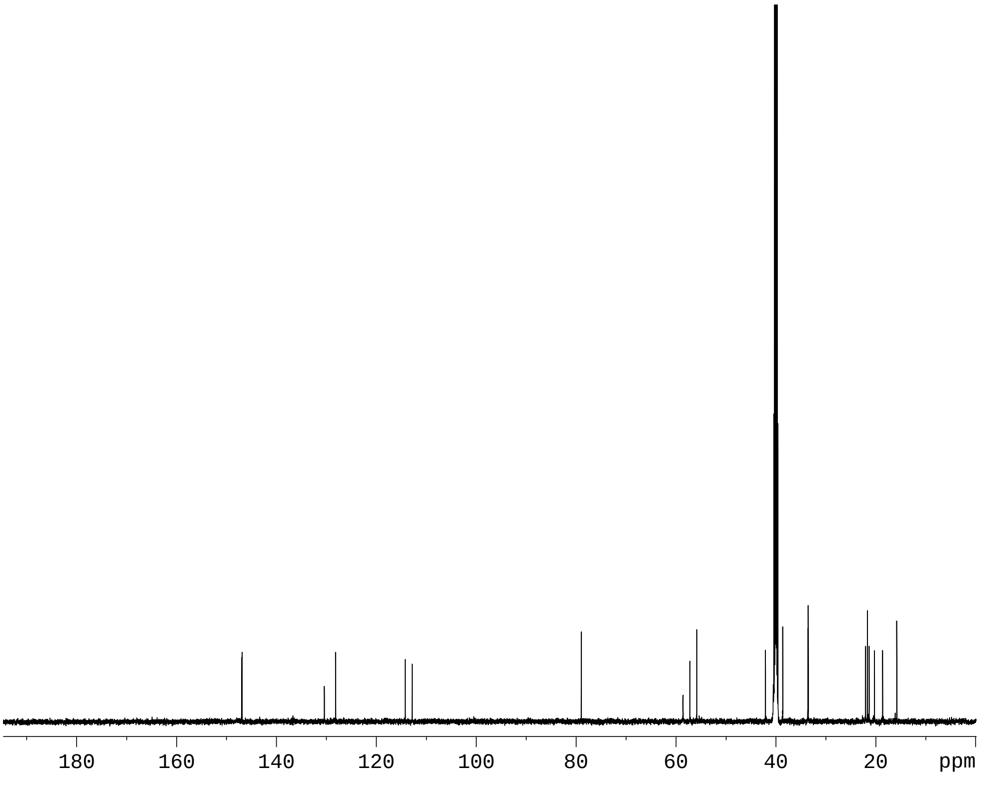

Figure S22: 1D ^{13}C NMR spectrum of cyclosiphonodictyol A (**5**) in DMSO-d_6, 303 K, 150 MHz.

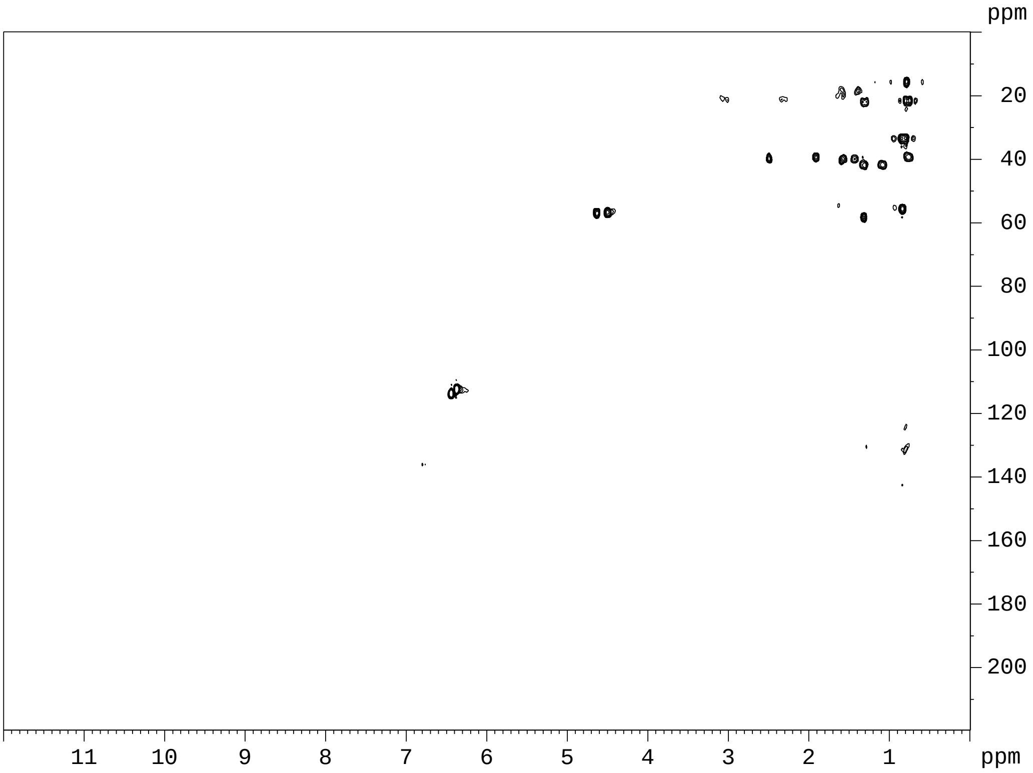

Figure S23: 2D ^{1}H,^{13}C-HSQC spectrum of cyclosiphonodictyol A (**5**) in DMSO-d_6, 303 K.

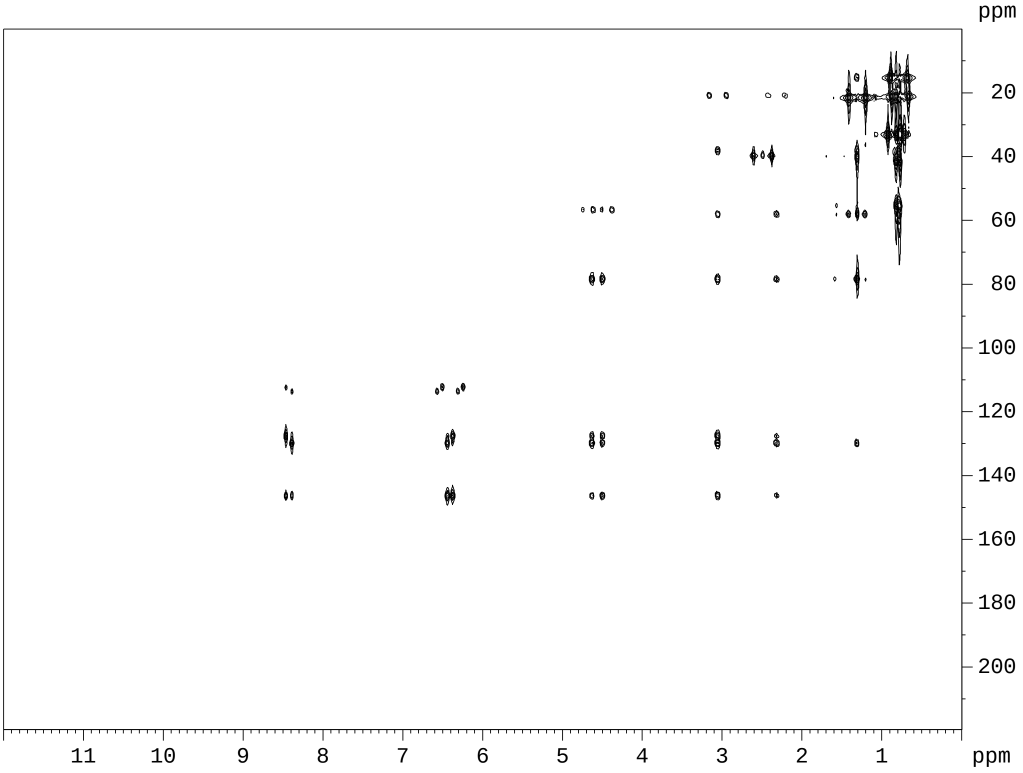

Figure S24: 2D ^{1}H,^{13}C-HMBC spectrum of cyclosiphonodictyol A (**5**) in DMSO-d_6, 303 K.

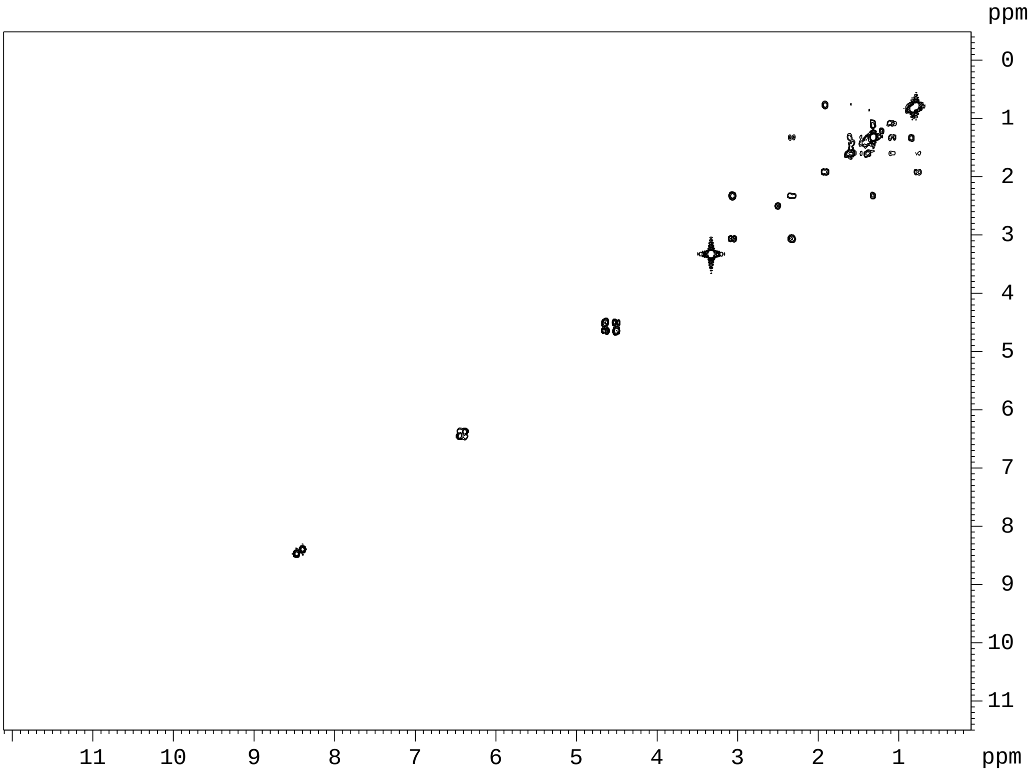

Figure S25: 2D ^{1}H,^{1}H-COSY spectrum of cyclosiphonodictyol A (**5**) in DMSO-d_6, 303 K.
